# Beyond Risk Prediction: Considering Upstream Universal Suicide Prevention to Decrease Risk and Increase Resilience

**DOI:** 10.3390/bs16020243

**Published:** 2026-02-09

**Authors:** Sarah Sparks, Cole Marvin, Regan Sweeney, Destiny Rojas, Sean M. Mitchell

**Affiliations:** Department of Psychological Services, Texas Tech University, Lubbock, TX 79409-2051, USA; sarah.sparks@ttu.edu (S.S.); cole.marvin@ttu.edu (C.M.); regan.sweeney@ttu.edu (R.S.); desrojas@ttu.edu (D.R.)

**Keywords:** suicide prevention, upstream prevention, universal prevention, prevention intervention, stress control, suicide ideation, suicide attempts, suicidal thoughts, suicidal behavior

## Abstract

Despite decades of research, suicide risk factors predict outcomes at chance levels, and there is a dearth of protective factor and resilience research, which limits the utility of risk-based approaches. Further, suicide prevention interventions primarily consist of individual psychotherapies and treating individuals after suicide-related outcomes occur. Unfortunately, there is a lack of upstream suicide prevention interventions targeting known suicide risk factors and aiming to increase well-being and resilience in the U.S. Thus, we discuss these problems in the field and the U.S. health care system and provide a possible solution. We propose using low-intensity, universal, and upstream prevention interventions, such as Stress Control. Stress Control is a classroom-style, Cognitive Behavior Therapy-based program shown to reduce “risk,” stress, anxiety, and depression and boost well-being and resilience as part of a stepped-care model. Although Stress Control’s suicide prevention effectiveness has not yet been directly assessed, we discuss how it could be a promising suicide prevention strategy with additional testing. A proposed mechanism for this reduction is building resilience to common risk factors and suicide ideation via evidence-based coping skills, thereby decreasing future suicide risk. We review current limitations and discuss how upstream, scalable, universal prevention interventions can help improve psychological resilience and reduce suicidal thoughts and behaviors, lowering the U.S. suicide rate. Implications and recommendations are discussed.

## 1. Introduction

Despite over 50 years of suicide prevention research (Fox et al., 2020; Franklin et al., 2017), suicide rates in the United States (U.S.) increased by 36% from 2000 to 2021, while worldwide suicide rates decreased (Centers for Disease Control and Prevention [CDC], 2023a; Martínez-Alés et al., 2022; Naghavi, 2019). In the U.S., suicide is a leading cause of death, with over 45,000 deaths in 2020 (National Institute of Mental Health [NIMH], 2022). Further, among U.S. adults, there are approximately 1.4 million suicide attempts per year, with over 710,000 individuals receiving medical attention following a suicide attempt (Centers for Disease Control and Prevention [CDC], 2020; World Health Organization [WHO], 2023). Rates of suicide ideation (SI) are even greater, with about 14 million U.S. adults reporting SI in the previous year (Centers for Disease Control and Prevention [CDC], 2023a). These statistics demonstrate the importance of continuing to focus on suicide prevention efforts.

Suicide prevention is best conceptualized as a public health problem, with multiple contextual factors impacting the development of SI and attempts. Overlapping social determinants of health, including legislation policies and commercial determinants, and individual risk factors, such as socioeconomic position or demographics, may increase poor mental health and suicide risk (Pirkis et al., 2024). For example, several social determinants of health have been associated with increased risk of psychiatric disorders and SI and suicidal behaviors (Na et al., 2025). Living and working in difficult, stressful, and unequitable circumstances can increase an individual’s stress, anxiety, and depression, which are suicide risk factors (Alegría et al., 2018; Alon et al., 2024; Howarth et al., 2020; Orsolini et al., 2020; Sareen et al., 2005). Public health suicide prevention programs are often focused on greater societal factors rather than clinical prevention interventions (e.g., Caine et al., 2018; Decker et al., 2018). For example, mental health suicide prevention interventions primarily include individual psychotherapy implemented after a point of crisis or suicide attempt (Brown & Jager-Hyman, 2014; D’Anci et al., 2019). Despite this, there is a gap in evidence-based public health interventions focused on universal mental health, suicide risk, and protective factors to improve well-being and resilience and decrease suicide-related outcomes for adults. 

Another factor that complicates suicide prevention is that current methods of predicting suicidal thoughts and behaviors remain only slightly better than chance (Franklin et al., 2017). Furthermore, the low base rate of suicidal thoughts and behaviors places mathematical constraints on positive predictive value (i.e., the likelihood that a model-identified positive case truly represents the phenomenon of interest) of tested models (Mitchell et al., 2021). This makes accurate prediction at the individual level very difficult, even with highly sensitive screening, stratification, and assessment strategies (Carter & Spittal, 2018). Even modern machine learning and health-system stratification models produce large area-under-the-curve statistics values but low positive predictive values (Belsher et al., 2019; Mitchell et al., 2021). Relying on risk prediction may miss suicide deaths or attempts and increase the burden on healthcare systems via false positive cases. 

There are also concerns about current interventions for suicidal thoughts and behaviors, specifically the uniformly small intervention effect sizes (Fox et al., 2020). Suicide interventions are limited in their reliability and ability to reduce suicide attempts or deaths (Fox et al., 2020). No treatment modality meaningfully outperforms another, and the modest effect sizes appear insufficient to counteract the limitations of prediction-based care (Fox et al., 2020). These converging limitations indicate that prediction-based methods do not reliably guide suicide-related clinical decision-making as a stand-alone strategy. Therefore, suicide prevention cannot hinge solely on identifying and treating a narrow “high-risk” group. 

Protective factors may also offer insight into how to further bolster suicide prevention efforts (Sher, 2019). Network analyses have revealed that self-esteem and social support had moderate strength centrality, while resilience, self-esteem, access to mental health services, and a positive attitude toward mental health services were inversely related to SI (Holman & Williams, 2022). Additionally, social support was associated with a decreased lifetime suicide attempt within U.S. and United Kingdom (U.K.) samples (Kleiman & Liu, 2013). Although limited, research consistently demonstrates that increasing protective factors may play a key role in suicide prevention efforts. 

More specifically, resilience may serve as a protective factor against suicide (Jeong & Noh, 2023; Roy et al., 2007). Resilience can be defined as the process of adapting well in the face of adversity, trauma, tragedy, threats, or significant life stressors (American Psychological Association, 2018). Individuals with high resilience possess several supportive factors and characteristics that reduce the risk of suicidal thoughts and behaviors, including engagement in activities and behaviors that strengthen social support, promote physical health, facilitate awareness of when external support is needed, and foster a sense of meaning in life (Levine, 2003; Sher, 2019). For example, integrating resilience into an intervention group for older adults significantly reduced SI and depressive symptoms compared to a control group (Zhang et al., 2022). However, there has been a lack of suicide prevention research focusing on resilience.

There is also a lack of *upstream* suicide prevention interventions to decrease psychological suicide risk factors and increase protective factors and resilience (McMahon, 2022). This is despite evidence that reducing psychological risk factors and increasing protective factors and resilience reduce SI and attempts (Elbogen et al., 2020; Sher, 2019; Turecki et al., 2019). Current suicide prevention efforts are disproportionately focused on treatment-oriented approaches, primarily individual psychotherapies delivered after suicide-related outcomes have occurred (Hofstra et al., 2020), rather than on prevention strategies aimed at enhancing well-being and resilience earlier in the risk trajectory (Centers for Disease Control and Prevention [CDC], 2022). This treatment-centric model is constrained by substantial barriers, including limited scalability amid global mental health treatment gaps of approximately 60–80%, poor access to services, clinician burnout, and a limited impact on population-level suicide rates in the United States (Alonso et al., 2018; Coombs et al., 2021; Fox et al., 2020; Hedegaard et al., 2020; McCormack et al., 2018; Thornicroft et al., 2017). Thus, implementing upstream suicide prevention interventions focused on increasing resilience, well-being, and protective factors prior to SI (i.e., *prevention interventions*) and interventions aimed at reducing the occurrence or reoccurrence of SI and attempts (i.e., *treatment interventions*; Centers for Disease Control and Prevention [CDC], 2022; Hofstra et al., 2020) may aid in a more holistic approach to suicide prevention. 

## 2. A Stepped Care Approach

One solution to the imbalance between need and available care is to implement stepped care that emphasizes true prevention interventions. Stepped care includes offering different types of interventions based on patients’ needs and preferences (Richards et al., 2012). In addition, stepped care can help address the current disparities in access to care for mental health and suicide prevention (Alvarez et al., 2022; Cook et al., 2019; Mohatt et al., 2021). This could be accomplished by incorporating evidence-based interventions that are easily disseminated to large numbers of people with minimal specialist services (Bower & Gilbody, 2005). Additionally, within this framework, there is greater capacity to provide early intervention and to triage patients to increasingly intensive interventions as needed, thereby facilitating timely, appropriate patient care (Bower et al., 2006). Evidence-based, low-intensity, suicide prevention interventions can also be utilized to target and reduce clinical risk factors for suicide and to increase protective factors and resilience, ultimately reducing SI and suicide attempts across a wider margin of the populace (Ki et al., 2024; Turecki et al., 2019).

Unfortunately, a major barrier to the effective implementation of prevention interventions within a stepped care framework is the lack of easily implementable suicide prevention interventions. One evidence-based, universal intervention that has been implemented in the U.K. to reduce depression, anxiety, and stress, and increase mental well-being and resilience for adults is *Stress Control*. Thus, we aim to reframe suicide prevention through a stepped-care lens, discussing Stress Control as a low-intensity, scalable, universal prevention intervention to enhance well-being and resilience and reduce suicide risk in the U.S.

## 3. What Is Prevention?

Prevention interventions for mental health aim to prevent the occurrence or development of disorders by promoting wellness, teaching coping skills, and providing psychoeducation through inclusive, wide-reaching programs (Goldsmith et al., 2002). Specifically, *upstream prevention interventions* focus on reducing factors that contribute to ill health and aim to prevent the onset of a disorder (McMahon, 2022). At the other end of the intervention spectrum, *downstream interventions* focus on individual behavioral change and treatments for established disorders (National Collaborating Centre for Determinants of Health, 2014). Although there are examples of upstream suicide prevention intervention efforts (e.g., peer support and group skill-building prevention interventions among United States Air Force [USAF] airmen; Wyman et al., 2020), most suicide prevention interventions focus on downstream interventions (e.g., treatment after suicide-related outcomes; Hofstra et al., 2020). 

**Figure 1:**
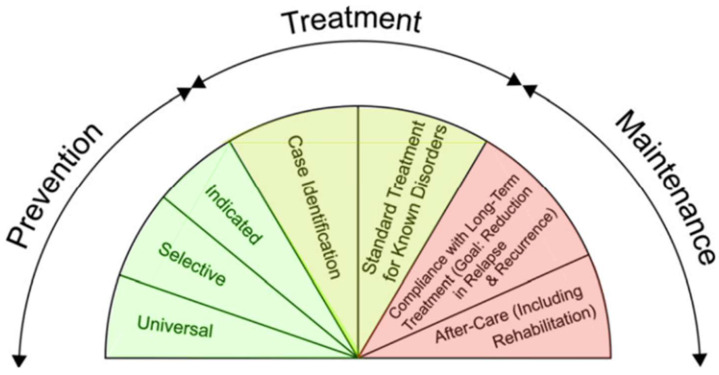
U.S. Institute of Medicine (IOM, 1994) Intervention Continuum. Note. A schematic of the U.S. Institute of Medicine’s intervention, or continuum of care, classification system. Highlighted in green are prevention interventions that primarily focus on equipping individuals with the skills and knowledge needed to reduce the likelihood of negative health behaviors. Highlighted in yellow are treatment interventions that provide individuals with clinical and supportive services, traditionally including inpatient, residential, intensive outpatient, or outpatient services. Maintenance, highlighted in red, provides individuals with a comprehensive long-term support network to prevent re-engagement in negative behavioral health outcomes. Used with permission from the National Academy of Sciences.

In addition to upstream and downstream interventions, the U.S. Institute of Medicine (IOM, 1994) further defines a spectrum of *prevention*, *treatment*, and *maintenance* interventions (see Figure 1). Prevention is subdivided into *universal* interventions, *selective* interventions, and *indicated* interventions. Treatment includes two subcategories: *case identification* and *standard treatment for the known disorder* (i.e., also includes interventions poised to reduce the likelihood of future co-occurring disorders). Maintenance includes *compliance with long-term treatment* (i.e., reducing relapse and recurrence of disorder) and *after-care* (i.e., rehabilitation services for the patient). 

An example of a similar, successful paradigm shift is evident in cardiovascular disease prevention (Knox et al., 2004; Ramôa-Castro et al., 2017). Cardiovascular research was focused on the prediction of patients with cardiovascular disease who would go on to experience myocardial infarctions and related deaths. Prediction rates mirrored current suicide prediction rates with a stagnation in patient outcomes despite substantial funds dedicated to cardiovascular disease research (Knox et al., 2004). However, a shift from treatment to prevention was key, including upstream and universal prevention interventions that encouraged physical activity, smoking cessation, and healthy diets among healthy individuals (Vogel et al., 2021). These universal prevention efforts successfully reduced myocardial infarction-related deaths and prevented cardiovascular disease in healthy individuals, resulting in a decrease in mortality between 1970 and 1990 (Knox et al., 2004). Following this example, expanding universal and upstream suicide prevention intervention efforts to include individuals who have not yet developed SI is one solution to deficits in risk identification, outcome prediction, and limited access to effective treatments (Coombs et al., 2021; Fox et al., 2020; Franklin et al., 2017). 

### 3.1. Stratified Suicide Screening and Interventions

**The Limitations of Screening and Risk Stratification as a Path to Suicide Prevention.** Within healthcare settings, identification of suicide risk factors and risk stratification are primary methods for allocating suicide prevention treatment interventions to patients (Large et al., 2017; Large & Ryan, 2014). However, suicide risk screenings often result in clinicians designating patients as “high, medium, or low” risk for suicide, despite screening measures having poor prediction validity of future suicide (e.g., Carter & Spittal, 2018; Large et al., 2017). *Targeted (i.e., also indicated/selective) screening* serves a purpose like case identification in suicide prevention. Targeted screening is conducted after a patient discloses clinical markers that warrant further evaluation (e.g., SI disclosure; Bryan et al., 2023a). The U.S. Preventive Services Task Force (2023) and current evidence-based suicide prevention recommendations support targeted screening in settings with low base rates of suicide-related outcomes, such as primary care (Bryan et al., 2009, 2022). 

*Universal screening* assesses all patients for SI at every healthcare visit (Goldstein-Grumet & Boudreaux, 2023). A benefit of this approach is the potential to identify individuals at risk who might not be identified through targeted screening (Boudreaux et al., 2016; Cui & Fiske, 2021; De Vylder et al., 2019; Raue et al., 2014). Recent evidence supports the use of universal screening and outreach in higher-risk settings, such as emergency departments and specialty mental health services (Mann et al., 2021). However, there is insufficient evidence to support universal suicide risk screening in low-risk settings (Bryan et al., 2023b; Nestadt et al., 2020; O’Connor et al., 2013). In low-risk settings, universal screening may increase the risk of false positives among patients who are screened as high risk (Bryan et al., 2023b). 

Importantly, neither targeted nor universal screenings will increase the identification of suicide risk among patients who do not wish to disclose risk. Up to 50% of patients may choose not to disclose SI or suicide attempts for various reasons, including stigma, potential repercussions (e.g., negative impacts on careers), or confidentiality concerns (Bryan & Morrow, 2011; Calear & Batterham, 2019; Husky et al., 2016; McGillivray et al., 2022; Sheehan et al., 2019).

Finally, suicide risk screenings are only able to identify patients who are already involved in the healthcare system. More than 12% of U.S. residents lack health insurance, and 44% of U.S. adults report difficulty paying for health care (Centers for Disease Control and Prevention [CDC], 2023b; West Health, 2022). Thus, a significant number of individuals may not regularly attend healthcare appointments or engage in indicated treatments due to financial and other barriers (Horwitz et al., 2020; Snow et al., 2019). Although research supports implementing both targeted and universal suicide risk screenings within healthcare systems, a more pressing concern is whether adequate services exist for those at risk (Mongelli et al., 2020). Upstream and universal prevention interventions may address these concerns.

**Standard Downstream Treatments for Identified Suicide Risk.** Current suicide prevention efforts exist within a stratified model and rely heavily on identification via screening measures and subsequent high-intensity treatment interventions, targeting individual factors (Fox et al., 2020). A significant limitation of psychotherapy treatments is reduced availability or access to mental health services (Coombs et al., 2021; David-Ferdon et al., 2016). Due to limited scalability (i.e., individual- or small-group formats), the number of patients that licensed providers can treat is severely restricted (David-Ferdon et al., 2016). Clinicians (e.g., psychologists, psychiatrists) who are appropriately trained in evidence-based suicide prevention treatments and best practices are even fewer, with wide variability in comfort, avoidance, and perceived self-efficacy amongst providers engaging in evidence-based practices with patients at elevated risk for suicide (Mitchell et al., 2020; Roush et al., 2018; Silva et al., 2016). Moreover, access to treatments is curtailed by barriers, such as stigma, discrimination, distrust of mental health providers, geographic locations (e.g., rural populations), and increasing financial costs for patients (David-Ferdon et al., 2016; Drapalski et al., 2008; Jensen et al., 2020; Rowan et al., 2013; Thornicroft, 2008). 

Ultimately, a predominantly downstream, stratified treatment model results in poor access overall, where only a minority of patients receive psychotherapy, and most receive no treatment (Bebbington et al., 2000; Lovell & Richards, 2000). This approach may explain, in part, why suicide prevention has fallen behind other public health problems and evidence-based, downstream treatment interventions, which have had a limited impact on suicide-related outcomes (David-Ferdon et al., 2016; Klonsky et al., 2020).

### 3.2. Applying a Prevention Framework to Suicide-Related Outcomes: Indicated, Selective, and Universal Prevention Interventions

*Indicated prevention interventions* for suicide prevention would be designed to target specific individuals who are exhibiting early signs of suicide potential but have not yet experienced SI within the population, enhancing protective psychological factors (e.g., coping and belonging) and resilience, as opposed to reactionary processes (Goldsmith et al., 2002; Pietrzak et al., 2011). In prior prevention interventions, such as the Prevention of Suicide in Primary Elderly Collaborative Trial (PROSPECT), depression care managers acted as a liaison between patients and primary care physicians, monitored treatment adherence, provided psychotherapy for depression when requested, and coordinated specialist referrals (Bruce & Pearson, 1999). This indicated that the prevention intervention was more effective than treatment as usual at preventing SI and reducing hopelessness (Reynolds et al., 2001). Similarly, recent reviews have confirmed the continued benefit of indicated prevention interventions, such as antidepressants, problem-solving-focused interventions, community support initiatives, or interdisciplinary care collaboration, to manage present mental health symptoms before they progress to SI in populations at elevated risk (e.g., adolescents and older adults; Sakashita & Oyama, 2019; Xavier et al., 2019). 

*Selective prevention interventions* would target at-risk groups regardless of their current mental health status, focusing on groups that have a higher likelihood of developing SI and aiming to prevent the onset of suicide-related outcomes among specific subpopulations (e.g., military members; IOM, 1994; Pruitt et al., 2019). Such interventions, such as gatekeeper trainings for community members (i.e., Question, Persuade, Refer [QPR]), emphasize the identification and referral of at-risk individuals to higher levels of care dependent on need, and have shown to minimize subsequent hopelessness and pessimism leading to escalation to suicidal behavior (Sakashita & Oyama, 2019). Another example is the USAF Suicide Prevention Program (AFSPP) delivered to USAF members. AFSPP is a multi-level suicide prevention program (e.g., involves leadership, education, and community preventive services; Knox et al., 2003). Data from 1990 to 2002 showed a reduction in the proportion of suicides each year across the decade, with rate reductions beginning when AFSPP was first fully implemented (Knox et al., 2003). Despite these promising decreases in suicide rates, the Air Force has experienced a recent increase in suicide rates (U.S. Department of Defense, 2020), suggesting that multi-level suicide prevention programs, although efficacious, address suicide risk better for some individuals than others. 

*Universal suicide prevention interventions* would address an entire population (e.g., nation, state, local community) regardless of individual symptoms or risk (IOM, 1994). These prevention programs would be designed to influence everyone, reducing suicide risk by removing barriers to care, enhancing knowledge of suicide prevention techniques, increasing access to help, and strengthening protective processes like social support, resilience, and coping skills (Goldsmith et al., 2002; Sakashita & Oyama, 2019). Importantly, preliminary studies have emphasized protective factors (e.g., social support, resilience) in intervention research aimed at preventing SI and related behaviors by enhancing coping skills for daily stressors (Allen et al., 2022; Antonio et al., 2020). Furthermore, increased social support, adaptive responses to fear, physical fitness, cognitive flexibility, and living in accordance with one’s values are associated with greater resilience, which reduces stress-related disorders and, in turn, suicide risk (Sher, 2019). The Wingman-Connect Program (W-CP), also implemented by the U.S. Air Force, is an example of a universal *prevention* intervention designed to address the needs of healthy and vulnerable service members simultaneously (Wyman et al., 2022). W-CP provided group trainings focused on protective factors against suicide, including social connectedness, social support, and effective stress management (de Aguiar et al., 2022; Dempsey et al., 2021; Leahy, 2017; Pickering et al., 2018; Rugo et al., 2020; Wyman et al., 2019). Despite its success in decreasing suicide deaths among military personnel, very few contemporary universal suicide prevention interventions designed for a general adult population have been widely implemented or empirically investigated.

Increasing resilience is a proposed mechanism for suicide prevention among those experiencing heightened risk factors to reduce subsequent suicide risk. Resilience-targeted interventions have shown reduced SI and depressive symptoms among older adults compared to a control sample (Zhang et al., 2022). A systematic review found that among 70 studies, resilience was one of the most valuable protective factors for reducing suicidal thoughts and behaviors among older adults (Ki et al., 2024). Introducing resilience-focused interventions throughout community care settings that both reduce risk factors and focus on maintaining well-being throughout life changes has the potential to mitigate the overall frequency of suicidal crises. In principle, any prevention intervention that targets known suicide risk factors, bolsters protective factors, increases suicide resilience, and promotes well-being could be considered a cog in the machine of universal prevention intervention for suicide prevention. 

A comprehensive approach to suicide prevention must combine upstream, universal prevention strategies to promote adaptive decision-making, wellness, and resilience among the entire population regardless of risk, selective and indicated prevention strategies supporting those at an increased risk, and treatment strategies designed to support individuals currently in crisis or at high-risk (e.g., active SI, recent attempt; Turecki et al., 2019). By expanding the conceptualization of suicide prevention to include upstream universal interventions, suicide prevention may experience a similar success in patient outcomes that has been seen in cardiovascular disease prevention and other public health efforts. One potential solution is an easily disseminable, pre-packaged, evidence-based program, such as Stress Control.

## 4. Stress Control 

Stress Control was developed as a low-intensity, high-volume intervention to increase access to mental health services in primary care without requiring a provider referral (e.g., J. White, 1995, 1997; J. White & Keenan, 1990). It has been embedded within the U.K.’s National Health Service (NHS) for over 40 years as part of a broader stepped-care model, functioning primarily as a guided self-help intervention that bridges the gap between individual psychotherapy and brief, single-session interventions, thereby reducing strain on the mental healthcare system while still addressing common mental health problems (J. White, 2022; J. White et al., 2008). Stress Control has been adapted for use by students, teachers, and parents in school settings, for online implementation, and has shown to function well within a community-based setting (K. White & White, 2021; J. White, 2017). It is a self-referral, didactic, psychoeducational program designed to be “low contact-high volume” (i.e., one professional to many attendees) to help alleviate provider shortages resulting from the proliferation of individual psychotherapies (David-Ferdon et al., 2016). Specifically, the use of psychoeducation, shown to be an effective evidence-based practice across mental health disorders; (Lukens & McFarlane, 2004) and coping techniques (e.g., diaphragmatic breathing; Hopper et al., 2019) for comorbid common mental health problems mirrors current transdiagnostic treatment models, like the Unified Protocol (UP; Barlow et al., 2016). For example, interventions targeting affective factors rather than disorder-specific symptoms have been effective in individual treatment and group settings, while seamlessly tying into stepped care approaches (Bullis et al., 2015; Ellard et al., 2010; J. White et al., 2008). 

Stress Control itself comprises 6 distinct yet interrelated, weekly, 90 min cognitive–behavioral therapy (CBT)-based psychoeducation classes (see the Online Supplementary Materials document and Table 1 for descriptions of each class; J. White, 1997; J. White et al., 2010b). Class sizes can be adjusted to accommodate a wide range of attendees (e.g., 6–160 individuals; Dolan et al., 2021; J. White et al., 2010b), increasing timely access to care and reducing service waitlists. Ideally, classes are offered on a cyclical basis throughout the year (e.g., session 6 is followed by session 1, repeating every 6 weeks); however, this schedule is flexible and dependent on local resources. The program is designed for attendees to complete classes sequentially, but this is not required (J. White et al., 2010b). For example, attendees can join Stress Control at any point in the program sequence, attend missed classes in a subsequent offering of the course, or repeat classes to reinforce their skills. 

Given the didactic nature of Stress Control, trainers are not required to be licensed mental health practitioners. For instance, Stress Control has been delivered by nurses (J. White et al., 2010a) and community members (J. White, 2022, personal communication) following a two-day in-person or virtual training provided by Dr. White and his team. This helps alleviate mental health provider shortages and burdens and provides a much-needed influx of evidence-based services in areas with such shortages (Mills et al., 2016).

Stress Control aims to prevent anxiety, depression, and stress, and enhance mental well-being (e.g., coping skills, promoting resilience) in at-risk populations, to decrease symptoms of mild to moderate “emotional disorders”, and to prevent symptom relapse through teaching attendees how to “be their own therapists”. Importantly, Stress Control is not a group therapy as individual care is not provided, and personal disclosure is not as highly emphasized. Furthermore, it is not intended to replace individual psychotherapy. Instead, it is designed as a low-intensity, CBT-based psychoeducation intervention that can be attended alongside other interventions. 

In Stress Control, stress is described as a common experience, with a focus on effective coping rather than “curing” stress (e.g., J. White et al., 2010a, 2010b), emphasizing the importance of cultivating resilience-focused strategies rather than eliminating stress altogether. The use of lay terminology is deliberate, aiming to reduce stigma (i.e., a barrier to mental health care) and increase help-seeking (P. W. Corrigan et al., 2014; P. Corrigan, 2004). The use of normalizing language extends throughout the program, with attendees referred to as “students” rather than patients, and “trainers” or “coaches” rather than “therapists” or “clinicians”. This approach has increased attendance and reduced attrition in populations that typically underutilize services and is unique to Stress Control compared to other similar didactic stress-reduction interventions (Hayes et al., 2020). 

The acceptability and feasibility of Stress Control have been demonstrated in multiple practice-based studies and a meta-analysis (e.g., Burns et al., 2016; Dolan et al., 2021; Mills et al., 2016). Indeed, Stress Control is typically the sole intervention for approximately 98% of attendees in the U.K., and 66% of attendees preferred Stress Control to individual therapy (J. White et al., 2010b). From 2013 to 2020, 7793 U.K. residents participated in practice-based research for Stress Control (Dolan et al., 2021). Furthermore, a multi-site evaluation of practice data revealed that 4451 individuals attended Stress Control over a 2-year period (2013–2015) across 5 rural health services (Delgadillo et al., 2016). Within this broader sample, 96% of participants highly recommended the treatment, showed a 50% reduction in anxiety and depression, and maintained an attrition rate average of 31% (Burns et al., 2016). These findings demonstrate the broad appeal of Stress Control, supporting its potential in suicide prevention.

## 5. Stress Control as a Suicide Prevention Intervention

Within a suicide prevention stepped-care framework, Stress Control simultaneously follows the U.S. IOM (1994) framework of prevention, treatment, and maintenance. Specifically, Stress Control would be considered a prevention intervention for someone interested in learning more about mental health and wellness overall (i.e., universal prevention), someone who was experiencing elevated stress (i.e., selective prevention), and someone experiencing SI but before any suicidal behaviors (i.e., indicated prevention). Prevention of an individual transitioning from SI to suicidal behaviors would occur through the reduction in known clinical risk factors for SI and the implementation of known protective factors. Early prevention is especially important, given that greater treatment effectiveness is observed when the disorder is detected early (Chan et al., 2018; Colizzi et al., 2020). 

Importantly, Stress Control aims to increase resilience by equipping attendees with knowledge (e.g., definitions of stress, anxiety, and depression) and coping skills (e.g., practices related to mindfulness, physical fitness, sleep hygiene), allowing individuals more effective ways of navigating future stressful life events, thereby reducing suicide risk overall. Reviews of comparable stress-reduction prevention interventions suggest that improvements in common mental health problems and subsequent downstream effects on suicide risk may operate through strengthening said coping resources and shifting cognitive reappraisal about one’s environment away from ruminative processes toward more effective problem-solving styles, both risk factors for suicidal thoughts and behaviors (Brosschot et al., 2006; Franz et al., 2021). Notably, the final Stress Control session explicitly focuses on maintaining gains in well-being and resilience beyond program completion. 

Although direct treatment for suicide-related outcomes is the best practice for suicide prevention, timely access to evidence-based care remains a barrier to effective suicide-specific treatment interventions (Labouliere et al., 2021; Mental Health America, 2023; Turpin et al., 2008). Therefore, for individuals with co-morbid SI and depression, for example, without access to suicide-specific treatment interventions, Stress Control would be considered both a proximal treatment for anxiety, depression, and stress and a distal treatment for SI. 

There are no studies that directly assess Stress Control’s impact on SI or other suicide-related outcomes. However, Stress Control has been shown to reduce risk (i.e., violence toward self and others), as measured by the 34-item CORE Risk Outcomes Measure (CORE-OM; Evans et al., 2002), a widely used indicator of mental health treatment outcomes in U.K. populations. The CORE-OM risk domain includes items assessing self-directed harm and suicidal cognition (e.g., thoughts of self-harm or being better off dead), as well as harm toward others (e.g., physical violence or intimidation), with studies demonstrating significant pre–post reductions following the 6-week program in community (Cohen’s *d* = 0.38, *p* < 0.001) and vulnerable incarcerated samples (Cohen’s *d* = 0.66, *p* < 0.001; Evans et al., 2002). Although the evidence base remains limited, these findings suggest that Stress Control may be relevant for suicide prevention while simultaneously addressing common treatment barriers.

## 6. Current Limitations to Prevention Interventions

Although Stress Control offers considerable scalability and flexibility as a proposed universal upstream suicide prevention intervention, these advantages, like those of other preventive approaches, are accompanied by inherent practical tradeoffs. Stress Control is currently implemented primarily as a universal community-based intervention in the U.K. Such interventions require unique funding sources, infrastructure, and personnel support, which are not often observed in fragmented healthcare delivery systems such as those currently observed in the U.S. Typically, prevention interventions are most successful when implemented within large, organized systems with considerable oversight, heightened compliance among community members, and centralized funding mechanisms. More specifically, the logistics (e.g., setup and operation) of community-based prevention interventions within an inconsistent, limited, and chaotic referral system pose unique obstacles for patients, particularly because the system relies heavily on patients finding and enrolling in programs on their own. Further compounding these challenges, Stress Control and similar programs, despite their broad preventive reach, are not designed to replace clinical treatment pathways; thus, referral to higher levels of stepped care remains necessary. For this reason, in addition to the obstacles mentioned previously, community prevention intervention programs would, at least initially, rely on mental health professionals to implement them, increasing the potential burden on an already overstretched workforce. 

Despite the widespread use of Stress Control in the NHS, research examining its effectiveness is somewhat limited and varies in study quality (Burns et al., 2016; Delgadillo et al., 2016; Dolan et al., 2021; Van Daele et al., 2013). For example, Stress Control has primarily been evaluated in practice-based studies, with only two randomized control trials (RCTs) evaluating its effectiveness (i.e., Hayes et al., 2020; Kitchiner et al., 2009; Mills et al., 2016; Wong et al., 2016). Another limitation and area for future direction is the lack of longitudinal studies that assess the effectiveness of regular or multiple-session attendance over months or years (Dolan et al., 2021). Moreover, most studies have been conducted with British patients, limiting generalizability. Although one RCT was conducted in Hong Kong, given the U.K.’s strong influence there until 1997, even this study’s participants may be less representative of a Chinese sample overall (Wong et al., 2016). Given these limitations, it is paramount to increase the number of RCTs on Stress Control and to evaluate its effectiveness across a more diverse range of populations.

Looking at examples from other prevention intervention-based programs, the US AFSPP provides limited insight into potential issues in implementing universal prevention interventions, given its implementation within an organization with clearly defined cultural boundaries, expectations, and a strongly reinforced command hierarchy (Knox et al., 2003). Perhaps the largest limitations in translating results from these examples are a lack of generalizability to the U.S. populace, including how universal upstream approaches will be funded in a healthcare system based in the profitability of targeted intervention. This is made worse, in part, by a lack of prioritization of such research at the federal level. 

## 7. Ways Forward and Proposed Solutions

Overall, expanding suicide prevention research to include healthy and at-risk populations will help to identify and address upstream factors that influence the development of suicide risk, as well as advance suicide prevention research. Although a focus on symptomatic individuals is necessary for acute crisis resolution and stabilization, it is clearly not sufficient given the rise in suicide-related outcomes despite increases in suicide prevention research and funding (Fox et al., 2020; Franklin et al., 2017). Evidence-based treatments are vital for suicide prevention; however, a greater number of evidence-based universal prevention interventions are needed to address mental health suicide risk factors before SI develops. 

Further, given that Stress Control is an evidence-based low-intensity intervention shown to effectively reduce common mental health problems, incorporating Stress Control as part of stepped care for suicide prevention could help inform the understanding of a progressive model of healthcare and low-intensity interventions for suicide prevention simultaneously. These results could help to fill a significant gap in the suicide prevention literature and inform later RCTs for low-intensity interventions for suicide prevention. Indeed, experimental comparison of Stress Control to other universal prevention interventions, particularly for adults in a community setting, in decreasing suicide risk factors and increasing resilience could be particularly helpful and fruitful. Thus, research evaluating Stress Control as a universal prevention intervention has the potential to greatly increase understanding in suicide prevention research.

The implementation of Stress Control could help to address barriers to mental health services and greatly reduce the number of treatment-seeking individuals who are currently unable to receive mental health healthcare. Additionally, because Stress Control can simultaneously serve as a prevention, treatment, and maintenance intervention, there is a greater opportunity to intervene at all stages of suicide risk and to extend intervention to individuals in prodromal or nascent states. This alleviates an overreliance on clinical treatment models and greatly expands the margins for successful intervention time points, which could aid suicide prevention overall (David-Ferdon et al., 2016). 

Research is needed to examine the direct impact of Stress Control on reducing the development of SI, as well as its impact on suicide-related outcomes and resilience. For our current American Foundation for Suicide Prevention research grant, we are implementing a Stress Control RCT within a U.S. urban community among individuals with SI during the prior month to identify if this upstream, prevention intervention is effective in reducing clinical and psychological risk factors and suicide-related outcomes and bolstering resilience and well-being. We hope to gain a greater understanding of the feasibility of implementing an evidence-based low-intensity intervention within the U.S. Additionally, we hope this project will inform needed solutions to the steady rise in suicide risk factors, suicide-related outcomes, and the lack of available effective mental healthcare services. Additional work in this area will be key to understanding effective community-based suicide prevention interventions.

## 8. Conclusions

A greater emphasis on earlier, scalable prevention interventions that strengthen resilience to known risk factors may help mitigate risk trajectories, reduce the likelihood of crisis escalation “downstream,” and increase overall well-being. Shifting to “true prevention” interventions could reduce reliance on suicide risk identification and prediction. This remains necessary when providers are placed in a position of determining who is in greater need of services, a symptom of a fragmented U.S. healthcare system. 

Further, universal prevention interventions offer a unique advantage in that they can be delivered broadly across populations, engaging individuals at varying levels of suicide risk (e.g., no SI to elevated risk, current SI, or prior attempts) within a single framework, while still engaging in stepped care and traditional intervention treatment pathways when imminent risk is identified. By providing earlier upstream access to evidence-based support, such prevention interventions may also enhance resilience and well-being, shifting the primary intervention target from safety to bettering individual lives (Goldsmith et al., 2002). This is especially salient when considering inequities in current approaches (e.g., cost and availability of individual psychotherapy) in suicide prevention and proactively considering how to address additional risk burden (e.g., stress, anxiety, depression) due to social determinants of mental health (Kirkbride et al., 2024; Na et al., 2025; Pirkis et al., 2024). 

Substantial change is still required to realistically implement universal upstream prevention interventions in the U.S. Indeed, more research is needed on the effectiveness of such interventions, aiming to not only reduce suicide but also identify mechanisms to enhance resilience. To mitigate the rising suicide rates in the U.S., we need a paradigm shift, changing how evidence-based low-intensity interventions are delivered to individuals who would otherwise not have access to mental health care services or suicide prevention interventions, as well as expanding the use of upstream, universal suicide prevention interventions.

## Figures and Tables

**Table 1:** Stress Control Class Descriptions.

Class Number	Class Topic	Key Lessons	Actions Taught
Class 1	What is Stress?	Functions of the class, how Stress Control works	Think of ways to weaken mechanisms of stress
Class 2	Controlling Your Body	How our body affects our feeling, fight/flight	Limiting caffeine, increasing exercise, belly breathing, progressive relaxation
Class 3	Controlling Your Thoughts	How our thoughts affect our feelings	Challenging unhelpful thoughts, breaking stress up
Class 4	Controlling Your Actions	How our actions affect our feelings	Facing fears, stepping out of your comfort zone, problem-solving skills
Class 5	Controlling Panicky Feelings & Getting a Good Night’s Sleep	How our breathing affects stress, how our sleep affects our stress	Controlling breathing, combining prevention skills, sleeping tips
Class 6	Boosting Your Wellbeing	Understanding existing compared to thriving, value alignment	Steps towards wellbeing, increasing mindfulness, compassion, gratitude

Note. “Stress” is used as a broad term for distress, including mental health distress, when presenting this information to participants.

## Data Availability

No new data were created or analyzed in this study. Data sharing is not applicable to this article.

## Supplementary Materials

The following supporting information can be downloaded at: https://www.mdpi.com/article/10.3390/bs16020243/s1, We provide descriptions of each Stress Control class in the Online Supplementary Materials document.
