# Supplementary material for: Beyond Risk Prediction: Considering Upstream Universal Suicide Prevention to Decrease Risk and Increase Resilience
Source: Behav Sci (Basel). 2026 Feb 9;16(2):243. doi: 10.3390/bs16020243 (PMC12938543; doi:10.3390/bs16020243)
Supplement: Supplementary file 1 [file behavsci-16-00243-s001.zip › behavsci-4097089-supplementary.pdf]

**ONLINE SUPPLEMENTAL FILE**

# Beyond Risk Prediction: Advancing Upstream Universal Suicide Prevention to Decrease Risk and Increase Resilience

## Overview of the Stress Control Classes

CBT theory-driven components (e.g., thought identification, behavioral activation) are divided and taught throughout each of the six classes. Like CBT delivered in individual therapy, each Stress Control class follows a standard format (i.e., a brief review of the previous session and homework assignment, introduction of agenda and new material, psychoeducation, strategies, review/homework assignment, and preview of next session). Short booklets corresponding to each class are provided free of charge to supplement and reinforce information learned throughout the course (e.g., common mental health problems and self-assessments) and to include homework assignments (e.g., relaxation exercises, thought records). Weekly booklets are available in both print and digital formats, thereby increasing accessibility (e.g., limited access to computers or the internet).

*Class 1* (i.e., "What is stress?") serves as an introduction to the program and provides psychoeducation about common reactions to stress and detailed information about common mental health problems. Consistent with low-intensity interventions within stepped care, the class also provides a rationale for helping each student "become [your] own therapist" (i.e., reducing exclusive reliance on mental health professionals for symptom improvement). Although not included in any studies evaluating Stress Control, this approach may also help attendees increase autonomy in their recovery.

*Class 2* (i.e., "Controlling your body") discusses the link between stress and the body (i.e., "fight or flight"). During this session, the trainer explains the rationale behind the

bidirectional relation between stress and somatic responses (e.g., state anxiety). Students learn how recognizing and altering somatic responses can improve perceptions of stress (e.g., diaphragmatic breathing). Psychoeducation about depression, anxiety, and effective coping techniques (e.g., exercise, relaxation techniques) is also provided during this session.

*Class 3* (i.e., "Controlling your thoughts") explains the bidirectional relation between stress and maladaptive thoughts. During this class, attendees are introduced to thought recognition, thought appraisal, and generating alternative thoughts (i.e., cognitive restructuring).

*Class 4* (i.e., "Controlling your actions") covers the interplay of stress and behavior (i.e., behavioral activation). This class emphasizes the importance of confronting fears and the maladaptive nature of avoidance. Students learn effective problem-solving and how social factors may cause or maintain stress.

*Class 5* (i.e., "Controlling panicky feelings and getting a good night's sleep") begins with a CBT explanation of panic and how psychological and somatic experiences of panic propagate stress. The role of breathing (i.e., diaphragmatic breathing) and the use of previously learned relaxation skills and preventive techniques are reinforced in the first part of this class. The second half of the class explains how stress can contribute to poor sleep, how insufficient sleep perpetuates stress, and provides psychoeducation on sleep hygiene.

*Class 6* (i.e., "Boosting your wellbeing, tying it all together, and controlling your future") focuses on techniques to boost wellbeing to complement the work on reducing stress and provides ways for attendees to cope in the future and maintain progress. The course concludes with an emphasis on well-being as an important aspect of mental health, distinct from symptom reduction or remission.
